# Efficacy and safety of transarterial chemoembolization plus sorafenib in patients with recurrent hepatocellular carcinoma after liver transplantation

**DOI:** 10.3389/fonc.2022.1101351

**Published:** 2023-01-13

**Authors:** Xia Zhang, Lirong Cai, Jian Fang, Fengsui Chen, Fan Pan, Kun Zhang, Qian Huang, Yuju Huang, Dongliang Li, Lizhi Lv, Man Chen, Ruiying Yan, Yanhua Lai, Yonghai Peng, Zhixian Wu

**Affiliations:** ^1^ Department of Hepatobiliary Disease, the 900th Hospital of Joint Logistics Support Force, Fujian Medical University, Fuzhou, China; ^2^ Department of Hepatobiliary Disease, The Third People’s Hospital of Fujian University of Traditional Chinese Medicine, Fuzhou, China; ^3^ Department of Hepatobiliary Surgery, the 900th Hospital of Joint Logistics Support Force, Fujian Medical University, Fuzhou, China; ^4^ Department of Hepatobiliary Surgery, Xiang’an Hospital, Xiamen University, Xiamen, China; ^5^ Department of Transplantation, People’s Hospital of Guangxi Zhuang Autonomous Region, Nanning, China; ^6^ Department of Oncology, the 900th Hospital of Joint Logistics Support Force, Fujian Medica University, Fuzhou, China

**Keywords:** hepatocellular carcinoma, sorafenib, transarterial chemoembolization, liver transplantation, overall survival

## Abstract

**Objectives:**

To explore the benefit and safety of transarterial chemoembolization (TACE) in combination with sorafenib in patients with recurrent hepatocellular carcinoma (HCC) after orthotopic liver transplantation (OLT).

**Methods:**

In this multi-center retrospective study, 106 patients with recurrent HCC after OLT were included. Fifty-two patients were treated with TACE plus sorafenib (TS group) and 54 were treated with TACE alone (TC group). Primary and secondary endpoints including overall survival (OS) and progression-free survival (PFS), and safety were assessed.

**Results:**

The median OS (17 vs 10 months, *P*=0.035) and PFS (12 vs 6 months, *P*=0.004) in the TS group were longer than those in the TC group. On multivariate analysis, BCLC stage (HR [hazard ratio]=0.73 [95% CI, 0.27–0.99], *P*=0.036) and sorafenib medication (HR=2.26 [95% CI, 1.35–3.69], *P*=0.01) were identified as independent prognostic risk factors for OS. No severe adverse events related to sorafenib were noted in the TS group. Four patients discontinued sorafenib due to intolerance.

**Conclusion:**

TACE in combination with sorafenib is a feasible regimen to improve the survival with mild toxicity in patients with recurrent HCC after OLT.

## 1 Introduction

Globally, hepatocellular carcinoma (HCC) is the third most common cause of cancer-related deaths (1, 2). According to estimates, China accounts for more than half of all deaths attributable to HCC in the world. Owing to the insidious onset of symptoms, most patients with HCC have medium or advanced-stage disease at the time of diagnosis. The natural survival of HCC patients after diagnosis is typically shorter than 6 months (3, 4).

Orthotopic liver transplantation (OLT) is one of the optimal therapeutic options for end-stage liver disease and transplantable HCC (5). The technological advances in drug development have enabled post-OLT five-year survival rates of >75% (6, 7). However, the risk of HCC recurrence is the major concern in transplanted patients. Prior to the development of sorafenib (the recommended molecular targeted drug for HCC), the primary management of recurrent HCC included surgical resection and transarterial chemoembolization (TACE). Several studies have now demonstrated the survival benefit conferred by sorafenib in patients with post-OLT recurrent HCC (8, 9).

Sorafenib, an oral multiple-tyrosine kinase inhibitor, has been shown to be effective in advanced HCC in randomized clinical trials and several small retrospective studies (10–14). A meta-analysis showed that sorafenib plus TACE improved overall survival (OS), time to progression (TTP), and progression-free survival (PFS) in patients with advanced HCC (15–17). However, whether TACE plus sorafenib is a beneficial therapeutic strategy for patients with post-OLT recurrent HCC is not clear (18–20). In this study, we retrospectively analyzed the efficacy and safety of TACE in combination with sorafenib in patients with post-OLT recurrent HCC.

## 2 Methods

### 2.1 Study population

Adult (≥18 years) patients who had undergone liver transplantation (transplantation criteria included Milan and Hangzhou criteria) at the Dongfang Hospital, Xiang’an Hospital, and the People’s Hospital of Guangxi Zhuang Autonomous Region between January 2009 and December 2015 were screened for eligibility.

Transplantation criteria and immunosuppressants

Milan criteria (13): tumor diameter ≤ 5 cm in patients with single tumor; ≤ 3 tumor nodules, each ≤ 3 cm in diameter in patients with multiple tumors; no major vascular invasion and distant metastasis. For HCC patients that exceeded the Milan criteria, Hangzhou criteria, a system proposed by China Transplantation Society, was employed (21).

Hangzhou criteria: patients without macrovascular invasion who qualify either of the two: (a) total tumor diameter ≤ 8 cm; (b) total tumor diameter > 8 cm, with histopathologic grade I or II and preoperative alpha fetoprotein (AFP) level ≤ 400 ng/mL.

All operations in this study were OLT. The immunosuppressive regimen was steroid tacrolimus plus sirolimus for 3 months post-transplant followed by low-dose tacrolimus plus sirolimus.

The study inclusion criteria were: (a) patients diagnosed with intrahepatic recurrence after transplantation by medical imaging and serum AFP level; (b) recurrent tumor with at least one measurable intrahepatic lesion; (c) survival time ≥ 12 weeks; (d): Child-Pugh classification: A, B (scores ≤ 7); (e): ECOG score: 0–1; (f): patients who received TACE plus sorafenib after recurrence were included in the TS group. Patients who received only TACE were included in the TC group.

Propensity score matching: To reduce the selection bias, a propensity score analysis was employed to minimize imbalanced distribution of treatments and confounders. The treatments were set as the dependent variable and confounders that potentially affect treatment were set as independent variables; then propensity scores were calculated using the software program. One-to-one matching was performed based on the calculated scores to select a propensity score-matched cohort of patients from both groups to compare the outcomes.

The study procedures conformed to the ethical principles enshrined in the Declaration of Helsinki, and were approved by the ethics committees of all three participating hospitals. Owing to the retrospective study design, the requirement for informed consent of individual patients was waived off.

### 2.2 TACE and sorafenib treatment

Procedure for TACE: The celiac trunk was cannulated using a standard percutaneous 5 French catheter such as the hepatic duct (Cook, Bloomington, USA). Digital subtraction angiography was performed to ensure complete visualization of all tumor vessels. Selective catheterization of the right or left hepatic artery was achieved using a micro-catheter (Cook, Bloomington, USA). A super-selective approach involving tumor-feeding vessels was utilized to minimize the risk of TACE-induced hepatic failure. Oxaliplatin (50–100 mg) and epirubicin (10–20 mg) mixed with 10–20 mL lipiodol were infused within 10 minutes to minimize the side-effects of nausea, vomiting, pain, ototoxicity, nephrotoxicity, and neurotoxicity. In 5 minutes, fluoroscopy was performed to determine whether full embolization of the tumor was achieved. The patients underwent treatment under conscious sedation.

Sorafenib was prescribed orally starting from day 3 post-TACE at an initial dosage of 400 mg/day. The dosage of sorafenib was adjusted according to the patient’s tolerance. The adverse events of sorafenib were graded. If the adverse events were ≥ grade 3 without effective remission, the dosage was reduced to 200 mg/day to relieve the adverse events. The drug was discontinued if the adverse events (≥ grade 3) did not remit after dose-adjustment.

### 2.3 Data collection

Data pertaining to the following variables were collected: (a) demographic characteristics; (b) clinicopathological parameters including BCLC stage, the diameter and number of tumors, tumor encapsulation, immunosuppressive regimen, Child-Pugh classification, serum AFP level, infection with hepatitis B virus, cirrhosis, complete blood cell counts, urine test, stool test, and coagulation function. The duration of sorafenib medication, PFS, adherence, and death were recorded.

### 2.4 Endpoint assessment

The follow-up interval was 1–2 months for patients with unstable conditions (generally for the first 6 months) and 3 months for patients with stable conditions. Patients were evaluated using abdominal ultrasound, computed tomography (CT), and magnetic resonance imaging (MRI). Efficacy was determined using the modified response evaluation criteria in solid tumor (mRECIST). The adverse effects of deranged white blood cell count, neutrophil count, hemoglobin, and platelet count, and the occurrence of hand-foot syndrome, diarrhea, hypertension, and rash were recorded. The adverse events were graded according to the classification criteria for common adverse reactions (CTCAE 3.0) of the National Cancer Institute (NCI).

### 2.5 Statistical analysis

Baseline characteristics of patients in the two groups were compared using the Chi-squared test or Fisher’s exact test. Kaplan-Meier method was used for survival analysis and the between-group differences in OS and PFS were assessed using the log-rank and Breslow test. Multivariate analysis of patient survival was performed using the Cox regression model. *P* values < 0.05 were considered indicative of statistical significance. All statistical analyses were performed using the SPSS 19.0 software (Chicago, USA).

## 3 Results

**Figure 1:**
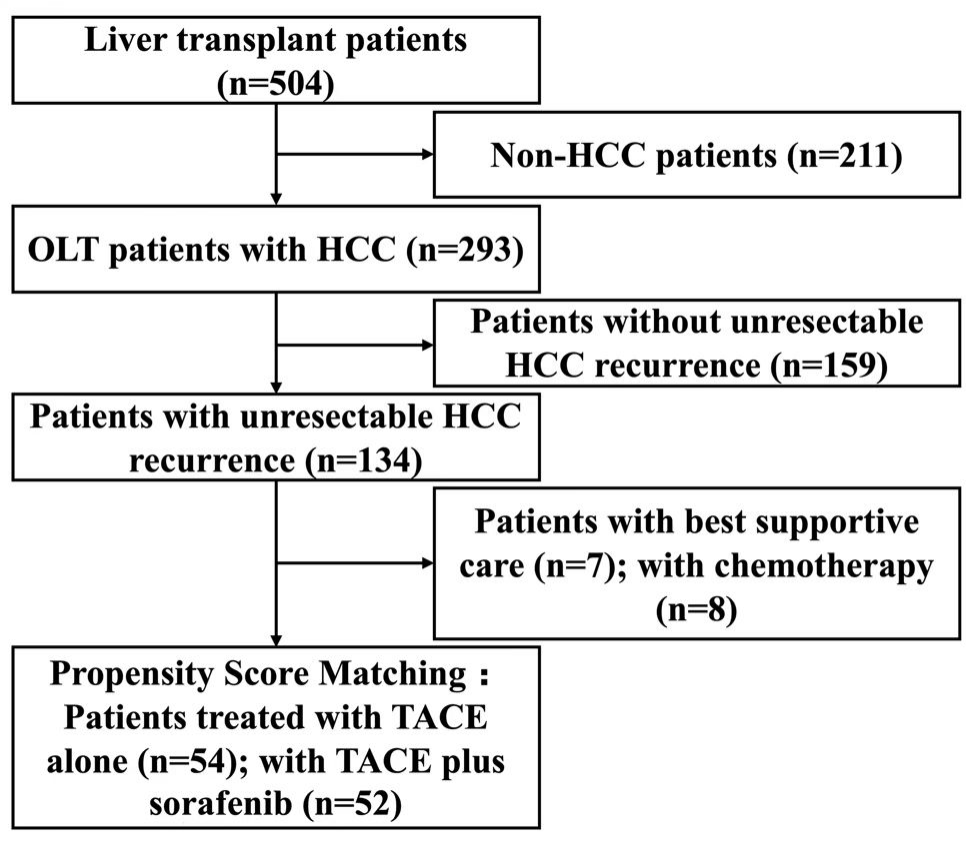
Schematic illustration of the study design and patient selection.

A total of 504 patients who had undergone OLT were screened. Of these, 293 patients had preoperative HCC. Among these, 159 had no recurrence or had resectable recurrence, and 134 patients had unresectable intrahepatic and/or extrahepatic recurrence diagnosed based on imaging findings and serum AFP level. Seven patients who received only best supportive care (BSC) and eight patients who received only chemotherapy were excluded. After propensity score matching, a total of 106 patients, including 52 patients treated with TACE plus sorafenib (TS group) and 54 patients treated with TACE alone (TC group) were included in this study. A schematic illustration of the study design and patient grouping are depicted in **Figure 1**.

### 3.1 Baseline characteristics

The baseline characteristics are presented in **Table 1**. There was no significant difference between the TS group and TC group with respect to sex (*P*=0.94), age (*P*=0.59), HBV infection (*P*=0.73), cirrhosis (*P*=0.74), serum levels of alanine aminotransferase (*P*=0.42), total bilirubin (*P*=0.43), white blood cell count (WBC, *P*=0.09), platelet count (*P*=0.13) and AFP (*P*=0.33), transplantation criteria (*P*=0.73), pathological grade (*P*=0.41), number of tumors (*P*=0.52), tumor diameter (*P*=0.32), encapsulation (*P*=0.92), BCLC stage (*P*=0.49), Child-Pugh grade (*P*=0.62), or immunosuppressant treatment (tacrolimus, *P*=0.50; sirolimus, *P*=0.67) (**Table 1**).

**Table 1:** Baseline characteristics of patients in the TS and TC groups.

Parameters	TS group (n=52)	TC group (n=54)	*P*
Sex			0.94
Male	46	48	
Female	6	6	
Age			0.59
<60	47	47	
≥60	5	7	
HBV infection			0.73
Yes	43	46	
No	9	8	
Cirrhosis			0.74
Yes	40	43	
No	12	11	
ALT (IU/L)	49.5 ± 11.3	47.8 ± 10.2	0.42
TBil (μmol/L)	13.6 ± 4.8	14.3 ± 4.2	0.43
WBC (×10^9^/L)	7.2 ± 1.3	6.8 ± 1.1	0.09
PLT (×10^9^/L)	152.8 ± 21.6	159.7 ± 24.3	0.13
Serum AFP (ng/mL)			0.33
≤400	24	30	
>400	28	24	
Transplantation criteria			0.73
Milan	20	19	
Hangzhou	32	35	
Pathological grade			0.41
Medium or low	42	40	
High	10	14	
Number of tumors			0.52
1	18	22	
≥2	34	32	
Tumor diameter			0.32
<5 cm	35	41	
≥5 cm	17	13	
Encapsulation			0.92
Yes	41	43	
No	11	11	
BCLC stage			0.49
A	7	5	
B	45	49	
Child-Pugh			0.62
A	35	36	
B	5	7	
Immunosuppressant levels			
Tacrolimus (ng/mL)	4.1 ± 0.7	4.2 ± 0.8	0.50
Sirolimus (ng/mL)	5.1 ± 1.2	5.0 ± 1.2	0.67
Time to recurrence (months)	9 (3–21)	11 (4–25)	0.58

### 3.2 Efficacy assessment

**Figure 2:**
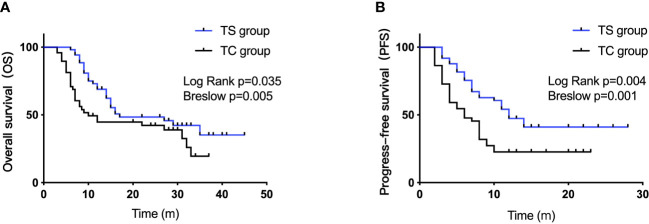
OS and PFS in the TS and TC group plotted by Kaplan-Meier method.

The median overall survival (mOS) was 17 months in the TS group versus 10 months in the TC group (log rank, *P*=0.035; Breslow, *P*=0.005, **Figure 2A**). The median PFS (mPFS) in the TS group and TC group were 12 months and 6 months, respectively (log rank, *P*=0.004; Breslow, *P*=0.001, **Figure 2B**). The 1-, 2-, and 3-year OS rates in the TS group were 68.9%, 48.4%, and 35.2%, respectively. The corresponding rates in the TC group were 44.71%, 42.2%, and 19.5%, respectively.

On univariate analysis, BCLC stage (HR=0.70 [95% CI, 0.16–0.98], *P*=0.022) and sorafenib medication (HR=2.94 [95% CI, 1.75–5.16], *P*=0.007) were identified as significant predictors of OS. On multivariate analysis, BCLC stage (HR=0.73 [95% CI, 0.27–0.99], *P*=0.036) and sorafenib medication (HR=2.26 [95% CI, 1.35–3.69], *P*=0.01) were independent risk factors for OS (**Table 2**). Age, male sex, AFP level, liver cirrhosis, tumor size, and time to recurrence were not identified as risk factors (*P*>0.05).

**Table 2:** Results of univariate and multivariate analyses showing prognostic factors for OS in patients with post-OLT HCC recurrence.

	Overall survival			
	Univariate		Multivariate	
	HR (95% CI)	*P*	HR (95% CI)	*P*
Age (<60 vs. ≥60)	0.93 (0.82–1.20)	0.65		
Male Sex	0.75 (0.33–1.34)	0.61		
AFP levels (≤400 vs. >400)	0.95 (0.77–2.19)	0.13	0.96 (0.84–2.34)	0.23
Liver cirrhosis	0.92 (0.62–1.82)	0.26		
Tumor size (<5 cm vs. ≥5 cm)	0.63 (0.14–1.41)	0.48		
BCLC stage	0.70 (0.16–0.98)	0.022	0.73 (0.27–0.99)	0.036
Sorafenib	2.94 (1.75–5.16)	0.007	2.26 (1.35–3.69)	0.01

### 3.3 Safety assessment

Adverse events considered to be caused by or related to sorafenib are listed in **Table 3**. The most common adverse effects were decreased hemoglobin (n=16, 30.8%), hand-foot syndrome (16, 30.8%), and rash (18, 34.6%) (**Table 3**). Other adverse events of sorafenib were leukopenia (14, 26.9%), thrombocytopenia (15, 28.8%), pruritus (12, 23.1%), hypertension (10, 19.2%), poor appetite (10, 19.2%), paresthesia (10, 19.2%) and alopecia (12, 23.1%). No level 4 adverse effects were observed. Sorafenib was discontinued in four patients.

**Table 3:** Adverse events in the TS group.

Adverse events		Incidence (n, %)			
	Total	Level 0	Level 1	Level 2	Level 3
Leukopenia	14 (26.9%)	1 (1.9%)	5 (9.6%)	5 (9.6%)	3 (5.8%)
Decreased hemoglobin	16 (30.8%)	5 (9.6%)	8 (15.4%)	1 (1.9%)	2 (3.8%)
Thrombocytopenia	15 (28.8%)	8 (15.4%)	4 (7.7%)	2 (3.8%)	1 (1.9%)
Hand-foot syndrome	16 (30.8%)	7 (13.5%)	4 (7.7%)	5 (9.6%)	0 (0%)
Rash	18 (34.6%)	4 (7.7%)	7 (13.5%)	6 (11.5%)	1 (1.9%)
Pruritus	12 (23.1%)	5 (9.6%)	3 (5.8%)	2 (3.8%)	2 (3.8%)
Hypertension	10 (19.2%)	4 (7.7%)	6 (11.5%)	0 (0%)	0 (0%)
Poor appetite	10 (19.2%)	4 (7.7%)	3 (5.8%)	3 (5.8%)	0 (0%)
Nausea	10 (19.2%)	3 (5.8%)	4 (7.7%)	3 (5.8%)	0 (0%)
Vomiting	9 (17.3%)	2 (3.8%)	5 (9.6%)	2 (3.8%)	0 (0%)
Diarrhea	12 (23.1%)	3 (5.8%)	6 (11.5%)	3 (5.8%)	0 (0%)
Paresthesia	10 (19.2%)	4 (7.7%)	3 (5.8%)	3 (5.8%)	0 (0%)
Alopecia	12 (23.1%)	3 (5.8%)	4 (7.7%)	5 (9.6%)	0 (0%)

## 4 Discussion

Sorafenib alone has been shown to be beneficial in OLT recipients with relapsed HCC (unresectable and not amenable to local treatment). In a retrospective study by Sposito et al., 15 patients with HCC recurrence after liver transplantation received sorafenib, and other 24 patients received BSC (13). The OS and PFS in the sorafenib-treated group were significantly longer than those in the BSC group (OS: 21.3 months vs. 11.8 months, respectively; PFS: 10.6 months vs. 2.2 months, respectively). In another retrospective study, patients with untreatable progression (including those who had undergone resection and local-regional treatment) were treated with either sorafenib or BSC; the median survival time in the sorafenib group was longer than that in the BSC group (14.2 vs. 6.8 months, respectively) (22). Additionally, Huang et al. found that in patients with primary hepatic carcinoma exceeding the Milan criteria, sorafenib reduced or delayed tumor recurrence after liver transplantation and improved patient survival with tolerable adverse effects compared with capecitabine (8).

The benefit of TACE plus sorafenib in OLT patients with relapsed HCC amenable to local treatment remains unclear. TACE in combination with sorafenib has been shown to confer a distinct advantage over single therapy in both single-center studies (23) and clinical trials with high level evidence (24–26); therefore, evaluating the efficacy of this therapeutic strategy in the setting of post-OLT HCC recurrence is of much clinical relevance. In this study, the median OS in the TS group was significantly longer than that in the TC group, which demonstrated the benefit of TACE plus sorafenib in this setting. Similar results were observed regarding mPFS. Additionally, there was a marked gap in the 1-, 2-, and 3-year OS rates between the TS group and TC group. Multivariate regression analysis suggested that the sorafenib medication conferred survival benefit. To the best of our knowledge, this is the first study to demonstrate that TACE plus sorafenib therapy may help improve the outcomes of relapsed HCC after OLT. Our findings suggest the need to conduct larger studies to provide more robust evidence. An accumulating body of evidence has demonstrated the benefit of sorafenib, either as monotherapy or in combination, in patients with recurrent HCC after liver transplantation. Interestingly, preoperative TACE plus sorafenib treatment was found to have a positive effect on the OS of OLT patients with preoperative unresectable HCC compared with TACE alone (27).

The adverse effects of sorafenib call for close monitoring. The major adverse events include myelosuppression, hand-foot syndrome, hypertension, gastrointestinal toxicity, and rash (11, 12). In the TS group, neutropenia, decreased hemoglobin, and thrombocytopenia were frequent. Symptomatic treatment has been shown to relieve the patient’s bone marrow suppression (12). Hand-foot syndrome, rash, pruritus, and hypertension were mild and were promptly relieved. In a study of 65 patients with recurrent HCC after liver transplantation, Kang et al. showed that 45 patients treated with sorafenib had longer survival compared to those treated with BSC, and that the toxicity was tolerable. Therefore, despite the adverse effects, sorafenib is recommended in this population and adverse events should be closely observed.

This was a retrospective study with a moderate sample size, which may have introduced confounders that resulted in bias. However, we performed propensity score matching to minimize bias caused by confounding variables. Because of the low incidence of post-OLT HCC recurrence, multi-center, prospective cohort studies are needed to investigate this issue more in depth.

## 5 Conclusions

In this study, patients treated with TACE plus sorafenib gained therapeutic benefit and exhibited acceptable toxicity. Thus, sorafenib targeted therapy provides an add-on alternative for patients with post-OLT recurrent HCC. A large randomized controlled trial is required to verify these findings.

## Data availability statement

The original contributions presented in the study are included in the article/supplementary material. Further inquiries can be directed to the corresponding authors.

## Ethics statement

The studies involving human participants were reviewed and approved by the Dongfang Hospital, Xiang’an Hospital, and the People’s Hospital of Guangxi Zhuang Autonomous Region. Written informed consent for participation was not required for this study in accordance with the national legislation and the institutional requirements.

## Author contributions

Conceptualization XZ, JF, LC, FC. Methodology FP, KZ. Validation QH, DL, LL. Formal analysis MC, RY. Investigation YL. Resources ZW. Data curation YP, YH. Writing—original draft preparation. ZW, XZ. Writing—review and editing ZW, XZ. Project administration FP, JF, KZ. All authors contributed to the article and approved the submitted version.
